# Phytomelatonin in Ornamental Horticulture: A Comprehensive Review of Growth Promotion, Stress Tolerance, and Post-Harvest Quality

**DOI:** 10.3390/ijms27041645

**Published:** 2026-02-08

**Authors:** Eman Abdelhakim Eisa, Andrea Tilly-Mándy, Péter Honfi

**Affiliations:** 1 Botanical Gardens Research Department, Horticulture Research Institute, Agricultural Research Center (ARC), Giza 12619, Egypt; 2 Department of Floriculture and Dendrology, Hungarian University of Agriculture and Life Science (MATE), 1118 Budapest, Hungary; tillyne.mandy.andrea@uni-mate.hu

**Keywords:** phytomelatonin, abiotic stress, ornamental horticulture, vase life, antioxidants, phytohormones

## Abstract

The ornamental plant industry faces escalating threats from erratic climate patterns and post-harvest perishability. Phytomelatonin (N-acetyl-5-methoxytryptamine) has emerged as a potent biostimulant capable of addressing these critical bottlenecks. This review synthesizes current knowledge on melatonin’s multifaceted roles in ornamental horticulture, clarifying the molecular pathways where it acts as both a direct Reactive Oxygen Species (ROS) scavenger and a signaling molecule orchestrating crosstalk with auxins, abscisic acid, and ethylene. We highlight applications in propagation, where melatonin synergizes with auxins to enhance rhizogenesis and promotes seed germination via hormopriming. Furthermore, we examine melatonin-mediated tolerance to abiotic stresses including drought, salinity, and temperature extremes emphasizing its role in preserving photosynthetic machinery and ion homeostasis. Crucially, the review addresses the post-harvest sector, demonstrating how melatonin extends vase life by repressing senescence-associated genes (SAGs) and antagonizing ethylene biosynthesis. Finally, we discuss future perspectives on genetic bio-fortification and commercial formulations, positioning phytomelatonin as a sustainable tool for securing the resilience and quality of ornamental crops.

## 1. Introduction

Encompassing cut flowers, turfgrass, and potted varieties, the ornamental plant market has evolved into a multi-billion-dollar global industry that is fundamental to urban landscaping, esthetic gratification, and environmental quality. Cut foliage, which contributes approximately US 1.4 billion to the floriculture economy, is a significant component of this sector, crucial for adding texture and depth to floral arrangements [1]. Nevertheless, the stability of horticultural output is under growing threat due to unpredictable global climate shifts. Horticultural plants are exposed to various forms of abiotic stress including extreme temperatures, heavy metals, drought, and salinity which can significantly impede their growth and productivity [2]. These non-living environmental factors pose a serious threat to agricultural sustainability, destabilizing ecosystems and reducing biodiversity [3,4,5]. Current estimates suggest that approximately 90% of arable land is now vulnerable to one or more of these stresses [6].

Among this diverse spectrum of environmental constraints, these major abiotic stresses are particularly concerning for the floriculture sector [7,8]. Regardless of the specific origin, these stressors impose convergent physiological bottlenecks that compromise the esthetic value and marketability of ornamental crops. These challenges are exacerbated by the demands of international trade and long-distance transport, which require careful handling to maintain quality and longevity during post-harvest stages [9].

Environmental stress factors fundamentally disturb the metabolic balance and water status within plant tissues. Whether driven by osmotic imbalance or thermal stress, these conditions lead to profound physiological impairments, including turgor loss, cell size reduction, and a marked decline in photosynthetic efficiency and nutrient uptake [10,11,12].

A critical commonality across these environmental constraints is the disruption of the cellular redox balance. Extended stress exposure induces a surplus of Reactive Oxygen Species (ROS), eventually overpowering the plant’s natural antioxidant defense systems. This surge in oxidation results in membrane injury, lipid peroxidation, and the breakdown of photosynthetic pigments, ultimately halting metabolic functions [13,14]. The major post-harvest issues that compromise the commercial value of cut foliage, such as *Danae racemosa* (ruscus), are leaf yellowing (chlorophyll degradation) and weight loss, which are driven by prolonged darkness, water imbalance, and the pivotal action of the plant hormone ethylene [15,16]. The need for sustainable alternatives to traditional post-harvest chemicals, such as those that inhibit ethylene action (e.g., silver thiosulfate), has become paramount due to environmental concerns [17].

In the search for sustainable biostimulants, Melatonin (N-acetyl-5-methoxytryptamine) has emerged as a revolutionary molecule. Although well-known as a neurohormone in animals, it was identified in the plant kingdom in 1995 and is now recognized as a versatile, multifaceted indolamine derived from tryptophan, with serotonin serving as a critical metabolic intermediate [18,19].

Unlike a simple growth hormone, melatonin acts as a “master regulator” with pleiotropic effects across various plant species [20]. It plays a vital role in regulating growth, development, and adaptability to adverse environmental conditions, functioning similarly to classic phytohormones [21]. Its primary mechanism involves acting as a potent antioxidant, directly scavenging ROS and improving ROS homeostasis by regulating the enzymatic antioxidant system [22]. Furthermore, melatonin has been reported to interact with other plant hormones such as ABA and ethylene to activate defense mechanisms under stress conditions and delay leaf senescence during abiotic stresses like heat, drought, darkness, salinity, and cold [23,24,25,26].

Recent applications have demonstrated its ability to enhance resilience against both drought and salinity by improving photosynthetic efficiency and promoting root development [27]. These findings, supported by studies on cut flowers like *Gerbera jamesonii* and tuberose, which show that melatonin enhances longevity, cell membrane stability, and antioxidant activity, position MT as a promising, eco-friendly post-harvest preservative [28,29].

This article presents the first comprehensive review specifically dedicated to the multifaceted roles of phytomelatonin in ornamental horticulture. We aim to synthesize current knowledge and critically examine phytomelatonin’s potential to overcome key industry bottlenecks from enhancing propagation rates and mitigating abiotic stress damage to extending the post-harvest vase life of cut flowers thereby positioning this molecule as a pivotal, sustainable tool for the modern floriculture sector.

## 2. Phytomelatonin: Biosynthesis, Signaling, and Molecular Mechanisms

Phytomelatonin, scientifically known as N-acetyl-5-methoxytryptamine C_13_H_16_N_2_O_2_ (Registry No. 73-31-4, CAS Common Chemistry; American Chemical Society), is an endogenous indolamine that exhibits a high degree of conservation among diverse evolutionary lineages [30]. Although originally discovered in the bovine pineal gland in 1954 [31], the presence of melatonin in vascular plants was independently confirmed by multiple research teams starting in 1995 [32,33,34,35]. This pivotal finding confirmed its widespread occurrence, leading to its identification in numerous crops, including maize [36], and grape [37]. The term “phyto-melatonin” was subsequently coined [38] to distinguish the plant-derived molecule from its animal counterpart [23].

### 2.1. Biosynthesis and Metabolic Pathways

The biosynthesis of melatonin originates from tryptophan, an essential amino acid, following biochemical routes that are preserved in both animal and plant systems [39]. The conversion pathway involves four main enzymatic steps:

1. Tryptophan Decarboxylase (TDC): Converts tryptophan into tryptamine, or tryptophan hydroxylase (TPH) and TDC convert 5-hydroxytryptophan into serotonin [40].
2. Tryptophan 5-hydroxylase (T5H): Facilitates the conversion of tryptophan into 5-hydroxytryptophan.
3. Serotonin N-acetyltransferase (SNAT): Transfers an acetyl group to convert serotonin into N-acetylserotonin, which is often considered a critical, rate-limiting step [41].
4. O-methyltransferases: The final methylation step is catalyzed by Acetylserotonin Methyltransferase (ASMT) or, uniquely in plant systems, Caffeic Acid O-methyltransferase (COMT). COMT can also methylate other substrates like caffeic acid and quercetin [41].

In plants, the generation of melatonin intermediates occurs across various subcellular compartments, including the cytoplasm, endoplasmic reticulum, chloroplasts, and mitochondria. While the canonical pathway of tryptophan → 5-hydroxytryptophan → serotonin → N-acetylserotonin → melatonin dominates in animals, plants often prioritize the tryptamine route (tryptophan → tryptamine → serotonin). Metabolites like 2-, 3-, and 6-hydroxy-melatonin confirm that melatonin is frequently not the final product in the plant system [42].

### 2.2. Physiological Functions and Signaling Networks

Melatonin (MT) functions as a vital and highly conserved indoleamine across the plant kingdom, acting not only as an antioxidant but also as a master growth regulator and signaling molecule [43]. Its efficacy in ornamental horticulture from propagation to stress tolerance stems from its intricate molecular mechanisms and its ability to integrate with the established hormonal network.

#### 2.2.1. Molecular Identity, Distribution, and Transport

Melatonin is present in nearly all plant species, with its concentration varying across tissues, developmental stages, and environmental light conditions [44,45]. Initially identified in species such as ivy morning glory (*Pharbitis nil* L.) [46,47], melatonin (MT) is especially abundant in ornamental plant families such as Rosaceae and Vitaceae. MT is transported through the xylem to different tissues, and evidence indicates active movement within intracellular organelles such as chloroplasts and mitochondria [23]. Because MT production is strongly influenced by photoperiod and circadian rhythms, accurately measuring endogenous melatonin requires careful consideration of plant age, seasonal variation, and the timing of tissue collection [48,49].

#### 2.2.2. Antioxidant Mechanisms: ROS Scavenging and Redox Modulation

**Figure 1:**
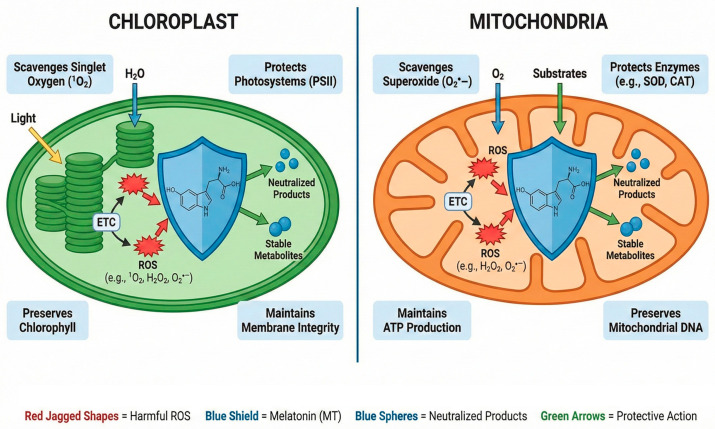
Melatonin (MT) acts as a dual-organelle antioxidant shield against Reactive Oxygen Species (ROS).

MT’s core defense function stems from its dual role as a direct scavenger and an enzymatic signal booster Figure 1, thereby enhancing cellular redox status under stress [50]. MT efficiently mitigates the excessive accumulation of Reactive Oxygen Species (ROS) and Reactive Nitrogen Species (RNS). The scavenging capacity of MT is remarkably high; reports indicate that it is five times more potent than glutathione (GSH) in *Salvia* sp. and fifteen times greater than mannitol [10,51].

This regulation is amplified through the MT-mediated stimulation of key redox enzymes, including Superoxide Dismutase (SOD), Peroxidase (POD), Catalase (CAT), and Ascorbate Peroxidase (APX) [52]. MT also modulates specific leaf senescence proteins that interact with photosynthesis regulation and macromolecules, influencing redox changes and the plant’s broader gene expression response to abiotic stress [53,54].

#### 2.2.3. Receptor-Mediated Signaling and Transduction Cascades

The elucidation of melatonin signaling receptors and pathways in plants represents a dynamic and evolving field of research. In the model plant *Arabidopsis thaliana*, the plasma membrane-localized protein CAND2/PMTR1, characterized by seven transmembrane domains, has been identified as a potential melatonin receptor [55]. Current signaling models propose that when melatonin binds to CAND2/PMTR1, it triggers the separation of the associated heterotrimeric G-protein α and βγ subunits (GPA1). It is hypothesized that this cascade activates the production of hydrogen peroxide (H_2_O_2_) via NADPH oxidase, subsequently regulating ion channels to induce potassium (K^+^) efflux and calcium (Ca^2+^) influx, ultimately leading to stomatal closure, a response abolished in CAND2/PMTR1 knockout mutants, supporting its role in melatonin perception.

However, this model is a subject of significant academic debate. Contradictory evidence challenges key aspects of the pathway, indicating that CAND2/PMTR1 may not be localized to the plasma membrane and might not be involved in activating melatonin-triggered defense gene expression [56]. Furthermore, research suggests that melatonin signaling can operate independently of the downstream Gα and Gβγ subunits. A central point of contention is the induction of H_2_O_2_; several studies report that melatonin application does not trigger H_2_O_2_ production in unstressed plants [57,58], a finding that conflicts with other reports [59]. Consequently, while CAND2/PMTR1 may function as a melatonin-binding protein, its role as a classical receptor and the precise nature of the downstream signaling components, particularly the relationship between melatonin and reactive oxygen species (ROS) dynamics, require further clarification [56,57].

At the molecular level, the transition from melatonin perception at the CAND2/PMTR1 receptor to physiological change is governed by a sophisticated signaling architecture. Beyond receptor binding, recent evidence suggests that melatonin triggers a MAPK (Mitogen-Activated Protein Kinase) cascade, specifically the MKK4/5-MPK3/6 module, which serves as a central hub for stress integration [11,23]. This activation leads to the transcriptional reprogramming of the cell via the up-regulation of WRKY, MYB, and NAC transcription factors. In ornamental species, this mechanism is essential not only for stress defense but also for the regulation of senescence-associated genes and the phenylpropanoid pathway, which influences stem structural integrity, floral pigmentation, and post-harvest longevity [34,45]. Furthermore, melatonin acts as a master regulator of calcium signaling by modulating the activity of calmodulin-like proteins that control stomatal behavior and cellular ion fluxes, thereby linking rapid environmental perception with long-term physiological adaptation [60].

#### 2.2.4. Hormonal Crosstalk: Integration with Phytohormone Networks

Melatonin (MT) interacts extensively with the major phytohormonal networks that regulate plant growth, development, and stress resilience. This crosstalk is especially relevant in ornamental species, where architectural traits, rooting behavior, and stress performance are key horticultural attributes.

The regulatory crosstalk between phytomelatonin and other phytohormones provides specific economic advantages in the ornamental sector that differ from those in agronomic crops. While crosstalk in cereals often targets harvest index and grain filling, in ornamentals, the MT–auxin interaction is a critical determinant of adventitious rooting efficiency, which is the cornerstone of clonal propagation for high-value woody and herbaceous species [61]. Furthermore, the antagonism between melatonin and ethylene/ABA is uniquely advantageous for maintaining ‘visual shelf-life’; by suppressing ethylene-signaling genes, melatonin delays the abscission of petals and leaves, directly preserving the esthetic marketability of cut flowers and potted plants under transport stress [62,63].

##### Auxins (IAA/IBA): The Rhizogenic Axis

MT displays notable auxin-like properties, largely attributable to its pronounced structural and functional similarity to indole-3-acetic acid (IAA). This resemblance confers considerable auxinic activity, enabling MT to participate directly in auxin-regulated developmental processes. In particular, MT modulates key components of the auxin signaling network, including receptors and downstream regulatory elements, thereby influencing the activation of pathways fundamental to root morphogenesis and vegetative propagation. Through this mechanistic crosstalk with endogenous IAA, MT contributes to the initiation and enhancement of root developmental programs essential for plant growth and propagation efficiency [64].

Melatonin demonstrates potent auxinic activity and serves as a key regulator of root morphogenesis. In the ornamental shrub *Lupinus albus*, melatonin application exhibited a 63% greater growth-promoting effect than IAA alone, underscoring its strong intrinsic efficacy [65]. This activity is underpinned by its capacity to enhance endogenous IAA biosynthesis, thereby increasing auxin availability in target tissues. Melatonin synergizes with classic auxins, including IAA, NAA, and IBA, to collaboratively promote the formation of both adventitious and lateral roots, establishing its role as a positive regulator of rooting in horticultural and ornamental crops [23,54,66]. Specific examples include significant improvements in root organogenesis in *Mimosa pudica* for vegetative propagation [60]. Furthermore, melatonin can exert precise, tissue-specific control, as evidenced in rice, where it restricts embryonic primary root elongation while simultaneously stimulating lateral root proliferation [61].

##### Cytokinins (CKs): Anti-Senescence and Thermotolerance

CKs are crucial for maintaining green tissues and delaying aging. MT acts as a stabilizer of this hormone, particularly under stress, and the two molecules exhibit mutual regulatory effects. Research on the MT–CK interaction indicates that exogenous melatonin treatment elevates endogenous CK levels. Reciprocally, CK has been observed to stimulate the expression of melatonin biosynthesis genes, consequently increasing endogenous MT concentrations. Furthermore, MT application results in the transcriptional upregulation of key CK signaling components, including response transcription factors (Types A and B ARRs) [58,62]. This synergistic relationship is evident in drought-stressed wild-type and isopentenyl transferase-overexpressing transgenic creeping bentgrass (*Agrostis stolonifera*). The combined action of CKs and MT leads to significant improvements in physiological indices crucial for stress tolerance, such as photochemical efficiency, chlorophyll content, and relative water content (RWC) [58]. MT enhances CK biosynthesis, which is vital for delaying leaf senescence and increasing heat tolerance. Under elevated temperatures, where CK levels typically experience rapid degradation, MT counteracts this decline by stimulating CK synthesis, thereby preserving ornamental quality and plant thermotolerance [43]. This mechanism is critical for turfgrass species like Perennial Ryegrass (*Lolium perenne*), where MT prevents heat-induced chlorosis [43]. The positive impact on longevity in cut ruscus foliage [63] is further supported by evidence that the inactive but readily convertible cytokinin storage form “trans-zeatin-O-glucoside (t-ZOG)” was significantly elevated in MT-treated foliage [67]. This observation suggests that MT activates cytokinin glucosyltransferases, thereby promoting cytokinin homeostasis and creating a reversible reserve pool of active CKs. Such regulation likely contributes to delayed leaf senescence and prolonged post-harvest quality.

##### Abscisic Acid (ABA): Drought Management

Melatonin acts as a critical modulator of Abscisic Acid (ABA) metabolism and signaling, making it central to plant drought-stress responses in ornamental species. The MT–ABA interaction is highly context-dependent, modulating ABA levels for optimal growth or defense. MT functions upstream of the ABA signaling pathway to regulate stomatal conductance. MT enhances drought resistance by significantly modulating key genes involved in ABA biosynthesis (MdNCED3) and degradation (MdCYP707A1/A2) [68]. In creeping bentgrass (*Agrostis stolonifera*), MT application under drought stress helps maintain higher relative water content (RWC) and turgor pressure through the modulation of ABA biosynthesis genes, thereby promoting efficient stomatal closure to minimize transpirational water loss [58,69]. This action supports the maintenance of shoot water content and reduces osmotic stress during severe drought [58]. Overall, MT improves drought tolerance by promoting cell elongation and elevating antioxidant activity [70]. In the context of the MT–ABA interaction, MT administration typically correlates with a reduction in ABA levels, often through transcriptional downregulation of ABA biosynthesis enzymes or altered ABA signaling sensitivity. Mechanistically, MT generally promotes the downregulation of 9-cis-epoxycarotenoid dioxygenase (NCED), a rate-limiting enzyme in ABA synthesis, and the upregulation of ABA catabolism genes (CYP707 monooxygenases), resulting in a rapid net decline in ABA concentration [71]. It is important to note that this response is strongly influenced by prevailing stress conditions and melatonin concentration. In some graminaceous species, including the ornamental grass Elymus nutans, the opposite effect of an increase in ABA levels has been observed [71]. A recent study on MT-treated cut ruscus foliage (*Danae racemosa*), a high-value ornamental green, demonstrated that MT significantly delayed leaf senescence and yellowing. This preservation was achieved through the suppression of abscisic acid (ABA) and ethylene levels, preventing the rapid decline in quality [63].

##### Ethylene: Post-Harvest Longevity and Senescence Control

Ethylene is recognized as the primary driver of senescence in many flowers and leaves, leading to wilting, color loss, and reduced vase life. MT acts as a strong inhibitor of senescence in ornamentals by controlling the ethylene pathway, although its effect is highly dependent on plant species and physiological stage. MT generally functions as a potent anti-senescence molecule by suppressing ethylene biosynthesis and signaling. Mechanistically, MT suppresses the expression of the key ethylene biosynthetic genes *ACS* (1-aminocyclopropane-1-carboxylic acid synthase) and *ACO* (1-aminocyclopropane-1-carboxylic acid oxidase), as well as genes related to cell wall degradation, including those encoding pectin-modifying enzymes [63,72]. This reduction in the autocatalytic rise in ethylene directly delays rapid senescence, wilting, and quality loss in cut flowers and foliage [73]. Consequently, post-harvest longevity is extended, and key ornamental attributes such as color and aroma are preserved. For example, treatment of cut ruscus foliage (*Danae racemosa*) with MT did not increase ethylene production, indicating that MT preserves foliage quality by maintaining a low ethylene status [63]. This modulation is particularly critical for maintaining the quality and vase life of high-value cut flowers such as carnation (*Dianthus caryophyllus*) and rose (*Rosa hybrida*) [74,75].

##### Gibberellins (GAs): Growth and Development

MT increases the levels of gibberellins (GAs) and helps to regulate the GA/ABA balance. By promoting GA accumulation [76], MT stimulates cell elongation and enhances growth parameters such as plant height and biomass—traits crucial for marketable quality during nursery production. MT treatment increases the transcript accumulation of GA biosynthetic genes. Studies have shown that MT stimulates growth by elevating GA levels and upregulating key GA biosynthesis enzymes, including GA20ox, GA3ox, and GA2ox [62]. MT also enhances GA sensitivity by upregulating GID genes (GA receptors). These genes encode the soluble GA receptor that forms a complex with GA and DELLA proteins, thereby preventing DELLA-mediated repression of GA signaling. This results in enhanced GA signaling and supports seedling growth [62]. This molecular support is highly relevant for ornamental crop production, where improved germination and vigorous seedling development are essential for an efficient nursery cycle [77].

#### 2.2.5. Comparative Advantages of Melatonin over Traditional Biostimulants

The emergence of melatonin as a “master regulator” in horticulture offers several strategic advantages over conventional biostimulants and synthetic growth regulators [11,20,35].

Environmental Safety and Sustainability: Unlike synthetic inhibitors like silver thiosulfate (STS), which pose heavy-metal contamination risks, MT is a natural, biodegradable indolamine with no known toxicity to humans or the environment [17,29,32].

High Potency and Economic Benefit: MT is exceptionally effective at micromolar concentrations, often requiring significantly lower application volumes than bulkier biostimulants like humic acids or seaweed extracts to achieve superior results [10,28,41]. Its dual role as both a direct ROS scavenger and a signaling molecule provides a higher efficacy-to-dose ratio, reducing overall input costs for large-scale nursery operations [51,52].

Low Labor Intensity: Melatonin’s high solubility and stability allow for seamless integration into existing nursery infrastructure [34,41]. It can be applied via standard foliar sprays, fertigation (root drenching), or as a simple additive in post-harvest vase solutions, requiring no specialized equipment or intensive labor beyond routine maintenance schedules [73,74,75].

## 3. Melatonin in Propagation and Development of Ornamental Plants

Melatonin application is instrumental in breaking seed dormancy and promoting germination through precise modulation of the abscisic acid (ABA) and gibberellin (GA) balance (Table 1).

GA/ABA Balance: Melatonin (MT) pretreatment critically regulates the hormonal balance in favor of germination. It promotes GA anabolism by upregulating the synthesis of GA_3_ in seeds, while simultaneously accelerating ABA catabolism through the induction of ABA metabolism genes (CYP707A family) and the downregulation of the ABA biosynthesis gene *NCED* [78]. This coordinated regulation significantly increases the GA_3_/ABA ratio, a hallmark of dormancy release.

Starch Catabolism: Melatonin also facilitates the mobilization of energy reserves essential for embryo growth. MT enhances starch degradation by increasing the activities of key starch-hydrolyzing enzymes, α-amylase and β-amylase, thereby supplying soluble sugars and energy required for hypocotyl elongation and radicle emergence; for example, in ‘Fengdan’ seeds, pretreatment with 100 µM MT significantly increased radicle thickness, an effect closely associated with enhanced starch catabolism and elevated synthesis of GA_1_ and GA_7_ [79].

Stress Germination: MT pretreatment significantly enhances the germination rate of sea lavender (*Limonium bicolor*) under salt stress [80]. This improvement is driven by a dual mechanism involving the rebalancing of phytohormones and the protection of essential metabolic processes.

Hormonal Crosstalk: MT directly modulates the GA/ABA ratio, which is critical for the release of seed dormancy. MEL promotes gibberellin accumulation by upregulating key GA biosynthesis genes, including *GA20ox* and *GA3ox*, resulting in increased levels of bioactive GA during germination. Concurrently, MT enhances ABA catabolism by downregulating ABA biosynthesis genes (*LbNCED1* and *LbNCED3*) while upregulating ABA degradation genes (*LbCYP707A1* and *LbCYP707A2*), thereby reducing the inhibitory effect of ABA on germination [81].

Metabolic and Cellular Protection: Salinity stress typically restricts seed germination by impairing nutrient mobilization and enzyme activity. MT pretreatment prevents the salinity-induced decline in amylase activity, preserving the function of hydrolytic enzymes responsible for starch degradation. This sustained enzymatic activity supports soluble sugar utilization and de novo protein synthesis, ultimately enhancing germination capacity [82]. In addition, MT maintains membrane integrity and stimulates antioxidant defenses, including superoxide dismutase (SOD) and peroxidase (POD), thereby alleviating oxidative damage and lipid peroxidation under high salinity [80].

**Table 1:** Differential physiological responses to melatonin application during seed germination and micropropagation.

Plant Species	Concentration Applied	Observed Effects on Rooting	Reference
*Prunus cerasus* × *P. canescens* (Cherry Rootstock)	0.5–1.0 mM	Foundational Study: Low doses (1 µM) increased root length (2.5×) and fresh weight (4×). High doses (5–10 µM) were inhibitory.	[83]
*Punica granatum* (Ornamental Pomegranate)	1.16 mg L^−1^ (equivalent to ≈5 µM)	Substitution Effect: The optimal concentration of MEL successfully substituted for IBA, achieving 100% rooting and high root counts (15.2 roots/plant). MEL also regulated the effects of H_2_O_2_ and GA_3_ on root morphology.	[84]
*Stevia rebaudiana*	5 µM, 20 µM, and 500 µM	Complex effects: 5 and 20 µM promoted germination only after 24 h dark pre-incubation. 500 µM was inhibitory to germination but most favorable for subsequent root development, promoting CAT and POD activity.	[85]
*Chrysanthemum morifolium*	100 mM	Stress Rooting: Improved root architecture and viability under drought stress, preserving root biomass.	[86]
*Mimosa pudica* L.	100 µM	Shoot Organogenesis Promotion: 70% explant response for shoot multiplication. Synergism: Response increased to 75–80% when combined with Ca^2+^ (5 mM). MEL action linked to Ca^2+^ channel activity.	[60]
*Lupinus albus* L.	0.001 to 100 µM	IAA-like Activity: MT induced the appearance of both lateral and adventitious root primordia from pericycle cells. It modified the number, length, and distribution pattern of roots, demonstrating a physiological effect similar to IAA as a root promoter.	[87]

### 3.1. Rhizogenesis (Rooting): Synergism in Woody Ornamentals

The efficiency of vegetative propagation via cuttings, a cornerstone technique for clonal multiplication of high-value ornamental varieties, is often constrained by the successful initiation of adventitious roots (ARs). Research conducted on the agricultural and ornamental species pomegranate (*Punica granatum* L. cv. ‘Wonderful’) has provided detailed insight into the molecular and hormonal cues governing this process, highlighting MT as a potent rhizogenic agent capable of enhancing adventitious root formation [84]. Melatonin, even at low concentrations, demonstrates strong efficacy in promoting adventitious rooting. An optimal application rate of 1.16 mg L^−1^ melatonin (MEL) was shown to effectively substitute for Indole-3-butyric acid (IBA), producing comparable improvements in rooting percentage and overall root system development. These findings indicate that MEL exhibits pronounced auxin-like activity in root induction and architecture formation, highlighting its potential as an alternative, eco-friendly rooting biostimulant in horticultural propagation systems [84].

Similarly, in Cherry rootstocks (often used for ornamental *Prunus* varieties), melatonin was found to alter the endogenous levels of IAA (Indole-3-Acetic Acid), promoting faster root primordia differentiation without the callus overgrowth often associated with high auxin doses [83]. Furthermore, melatonin also influences the regeneration of the lateral root system and the formation of adventitious roots in *Lupinus albus* seedlings, and it synergistically enhances rooting when applied in combination with indole-3-acetic acid (IAA) [87]. In Sensitive Plant (*Mimosa pudica*), often grown as a novelty ornamental, melatonin was confirmed to exhibit auxin-like activity, promoting extensive rhizogenesis. The study suggested that melatonin protects the cutting base from wounding-induced oxidative stress, creating a favorable redox environment for root initiation [60].

### 3.2. Vegetative Growth

Once propagated, the visual quality of ornamentals (leaf color, shoot compactness) determines market value. Melatonin significantly enhances vegetative biomass in landscape and nursery crops.

In Bermudagrass (*Cynodon dactylon*), a primary turf species for landscapes and golf courses, exogenous melatonin application significantly improved shoot height and biomass accumulation under stress. The mechanism involves the upregulation of photosynthetic capacity and the maintenance of leaf electrolyte leakage, ensuring greener, denser turf [88].

In Chrysanthemum (*Chrysanthemum morifolium*), melatonin application protected the photosynthetic apparatus (PSII) during heat stress, preventing leaf yellowing (chlorosis). By maintaining higher chlorophyll content and optimizing nitrogen metabolism, melatonin-treated plants exhibited superior leaf area and stem robustness compared to untreated controls [86].

## 4. Abiotic Stress Tolerance: Melatonin-Mediated Preservation of Esthetic Quality

In the ornamental plant industry, abiotic stress does not merely reduce yield; it degrades visual quality, manifesting as leaf chlorosis, wilting, necrosis, and flower abscission, effectively rendering the crop unmarketable. Phytomelatonin (MT) acts as a pleiotropic molecule, orchestrating a defense response that preserves both the physiological integrity and the esthetic value of plants under adverse environmental conditions (Table 2).

### 4.1. Osmotic Stress: Salinity and Drought

Drought and high salinity are primary limiting factors for landscape ornamentals and turfgrasses. Under drought and high salinity, melatonin (MT) application consistently enhances growth traits and gas exchange parameters by mitigating osmotic stress through two primary strategies: the regulation of ion transporters and the promotion of osmotic adjustment [105,106]. First, MT preserves the visual quality of ornamentals by maintaining the critical Na+/K+ balance under saline conditions, which is crucial for preventing the marginal leaf burn and necrosis that reduce esthetic value [107]. Mechanistically, MT achieves this by upregulating the expression of ion transporter genes such as Salt Overly Sensitive 1 (SOS1) and enhancing the activity of H+ pump activity like the vacuolar H+-ATPase, which limits the toxic accumulation of sodium ions [108,109,110]. Second, MT promotes the accumulation of compatible solutes, such as proline, glycine betaine, and soluble sugars, which aid in cellular osmotic adjustment [111]. This helps maintain cellular turgor pressure and prevent the wilting of delicate floral organs under drought [112]. These mechanisms are supported by MT’s broad protective effects, including a significant reduction in damaging Reactive Oxygen Species (ROS), Malondialdehyde (MDA), and Electrolyte Leakage (EL) through the upregulation of both enzymatic (SOD, CAT, POD) and non-enzymatic (AsA, GSH) antioxidant activities [113,114]. By scavenging H_2_O_2_ and O_2_^−^ [43,109], MT alleviates salt and drought-induced damage to Photosystem II (PSII) reaction centers and prevents chloroplast damage [115,116,117,118]. Together, these actions safeguard the root system architecture, enhance root activity, and stimulate vegetative growth under water deficit [12,119].

#### Gene Regulation

Genes related to MT biosynthesis, such as COMT (Caffeic Acid O-Methyltransferase), are implicated in alleviating salt stress. The upregulation of SlCOMT1 in tomato aided in minimizing ion damage, enhancing antioxidant enzyme levels, and improving the regulation of mineral nutrient fluxes [120,121]. Ectopic expression of the MT synthesis-related gene *SNAT* in *Arabidopsis thaliana* increased endogenous MT production in mitochondria, thereby reducing ROS-induced oxidative stress under drought [122].

Transcriptional Crosstalk: MT activates the (AP2/EREBP-HB-WRKY) transcriptional cascade [123] and interacts with various hormones and plant growth regulators, including zeatin, gibberellin A_14_, and jasmonic acid, to bolster tolerance to drought stress [124]. MT also regulates the balance between ABA and Gibberellic Acid (GA), which is essential for seed germination under stress [125].

### 4.2. Temperature Extremes: Mitigating Thermal Injury

MT plays a critical role in maintaining membrane fluidity and stability against chilling and heat damage, preserving esthetic quality. MT enhances the chilling tolerance of cold-sensitive ornamentals. In miniature rose (*Rosa hybrida*) and Bermudagrass (Cynodon dactylon), MT reduces lipid peroxidation (measured as MDA) by upregulating cold-responsive genes (CBFs) and antioxidant enzymes [126,127]. This action stabilizes the plasma membrane, preventing the transition to a rigid gel state that causes electrolyte leakage and chilling injury [88,128]. Heat Stress and Prevention of “Scorch”: In Chrysanthemum (*Chrysanthemum morifolium*), MT prevents heat-induced oxidative bursts. It enhances the biosynthesis of Heat Shock Proteins (HSPs) and stimulates the Ascorbate–Glutathione cycle, neutralizing the ROS generated by heat stress and preventing chlorophyll degradation that leads to leaf yellowing or “scorch” [92].

### 4.3. Heavy Metals and Waterlogging

MT extends its protective capacity to anaerobic stress and heavy metal toxicity, important for urban phytoremediation.

Heavy Metal Tolerance: MT enhances the survival of ornamentals in metal-contaminated soils through chelation and compartmentalization. In *Malva parviflora*, MT promotes the synthesis of phytochelatins (PCs), which bind metal ions (e.g., chromium) and transport these PC-metal complexes into the vacuole, sequestering toxins away from active cytoplasmic processes and reducing necrosis and stunted growth [129,130]. In tuberose flowers (*Polianthes tuberosa*), melatonin (MT) application effectively mitigated arsenic-induced stress, resulting in enhanced growth traits, improved chlorophyll content, and preservation of vase life [98].

Waterlogging Mitigation: Under anaerobic conditions, MT enhances SOD and POD activities in ornamentals such as clematis (*Clematis* sp.), reducing oxidative stress caused by waterlogging through lower H_2_O_2_ levels and decreased ethylene accumulation [93].

## 5. Melatonin in Flowering and Secondary Metabolism

Melatonin (MT) acts as a powerful signaling molecule in plant reproductive development, coordinating the transition from vegetative to reproductive growth and significantly enhancing the visual quality of flowers through the regulation of pigments and volatile compounds.

### 5.1. Regulation of Floral Transition

Melatonin functions as a chronoregulator, helping plants interpret environmental signals (like photoperiod and temperature) to initiate flowering at the optimal time. Recent research has identified a novel function for melatonin as a growth regulator in controlling flowering time [131]. Early studies, such as those disrupting signaling in *Chenopodium rubrum* leaves using electrical currents, suggested that alterations in endogenous melatonin rhythmicity could interfere with flowering [132], marking *C. rubrum* as the first plant species where MT’s circadian regulatory role was identified [133].

The effect of MT on floral timing is biphasic and dose-dependent, with evidence supporting both acceleration and suppression mechanisms:

*Acceleration Mechanism* (Via *FT*): MT can accelerate the floral transition, particularly under non-optimal photoperiods or stress. This is linked to the core photoperiod pathway, where MT increases the transcript levels of *FLOWERING LOCUS T* (*FT*), the central florigen protein, often mediated via the upregulation of the upstream gene *CONSTANS* (*CO*) [134]. This provides a valuable mechanism for controlling bloom time in commercial cut flowers.

*Suppression Mechanism* (Via *DELLA and Crosstalk*): Conversely, MT is also implicated in delaying the floral transition, a crucial regulatory function. Shi et al. [135] roposed a mechanistic model demonstrating that MT suppresses floral transition by stabilizing DELLA proteins repressors of the Gibberellin (GA) pathway, thereby disrupting transcription factors associated with flowering. This regulatory function has been corroborated by several studies [131,135]. Further evidence of suppression emerged from transgenic rice plants carrying the (*SNAT*) gene from sheep, where elevated MT levels significantly repressed flowering [42].

Recent investigations have also highlighted the complex interplay between MT and other signaling molecules:

*Strigolactones:* Zhang et al. [134] established a direct connection between (MT) and strigolactones (carotenoid-derived phytohormones). Their findings suggest that strigolactones function upstream of (MT), delaying flowering through the activation of (FLC) when (MT) levels exceed a specific threshold [134].

*Nitric Oxide* (*NO*): NO, whose synthesis is induced by MT, has been shown to increase DELLA protein levels [136] and repress flowering in *Arabidopsis* [137]. Shi et al. [135] reported a direct link between MT and floral transition, proposing that NO may contribute to MT-mediated stabilization of DELLA proteins, ultimately delaying flowering [135].

### 5.2. Pigmentation and Visual Quality

The intensity and stability of flower color are the most crucial factors defining the market value of an ornamental crop. MT enhances pigmentation by stimulating the necessary biosynthetic pathways.

Anthocyanins: MT acts as a metabolic trigger, upregulating the gene expression of key enzymes in the phenylpropanoid pathway, such as Phenylalanine Ammonia Lyase (*PAL*), Chalcone Synthase (*CHS*), and Dihydroflavonol 4-reductase (*DFR*) [138,139]. In both green and purple mustard genotypes, exogenous melatonin increases the expression of structural genes and the activity of key enzymes, resulting in higher anthocyanin accumulation and reduced oxidative damage. This mechanism effectively restricts Vanadium (V) deposition in tissues and prevents leaf chlorosis, highlighting a synergistic defense system where melatonin-mediated pigment production and restricted metal uptake preserve the plant’s physiological integrity [140].

Carotenoid Homeostasis: MT exerts a profound regulatory influence over plastidial function, a role crucial for stabilizing photosynthetic machinery and metabolite synthesis under stress. Mechanistically, as demonstrated in model systems exposed to excess light, MT mitigates photoinhibition by enhancing photosystem efficiency and orchestrating the expression of both nuclear- and chloroplast-encoded genes. This regulation primarily targets the chloroplast transcription machinery and housekeeping genes to maintain functional integrity rather than directly altering photosynthetic structural proteins, exhibiting a clear dose-dependent response where lower concentrations effectively balance chloroplast functions while high doses can prove inhibitory [141].

This preservation of plastidial integrity creates the necessary metabolic environment for pigment accumulation, a phenomenon clearly observed in African marigold (*Tagetes erecta* L.). In this species, exogenous melatonin application (200 µM) significantly elevates the levels of lutein and zeaxanthin, essential carotenoids that define floral color and commercial quality. By stabilizing these plastid-derived pigments and bolstering antioxidant defenses, including GABA and proline, melatonin mitigates the degradative effects of drought stress, ensuring the preservation of vibrant floral displays and enhancing the plant’s overall bioactive value [142].

### 5.3. Scent and Secondary Volatile Metabolites

Scent compounds, such as terpenes, are volatile secondary metabolites produced via the mevalonate MEV and methylerythritol phosphate MEP pathways [143]. Since MT is linked to the overall plant stress defense system, which often upregulates these pathways, it is hypothesized that MT treatment can indirectly enhance the production and emission rate of specific fragrance volatiles in ornamental species like White garland or ginger lily (*Hedychium coronarium*) [144]. Further targeted research is required to quantify the direct impact of MT on commercially relevant floral volatile profiles.

## 6. Post-Harvest Physiology and Vase Life

The cut flower industry’s profitability relies heavily on maximizing vase life, a trait highly sensitive to ethylene, water status, and microbial attack. Melatonin (MT) has emerged as a superior post-harvest dipping or vase-solution treatment (Table 3), often providing synergistic or complementary results compared to traditional chemical preservatives. MT directly improves marketable vase life (Category 1) by modulating senescence genes and stabilizing cellular structures [73].

### 6.1. Anti-Senescence

Floral and leaf senescence is a genetically programmed event. MT acts as a powerful anti-senescence agent by altering redox parameters and repressing cellular dismantling, complementing its role as an ethylene antagonist (detailed in Section 3.1).

MT mitigates oxidative stress by altering redox parameters, including reducing levels of Reactive Oxygen Species (ROS), increasing non-enzymatic antioxidants such as Ascorbate (AsA) and Glutathione (GSH), and enhancing the activities of antioxidant enzymes such as Superoxide Dismutase (SOD) and Catalase (CAT) [144,157].

In Peony (*Paeonia lactiflora*) cut flowers, MT significantly enhanced vase life by stabilizing cell membrane integrity and preserving chlorophyll content [153]. MT suppresses the molecular program of aging. MT treatment blocks the activation of several Autophagy-Related Genes (ATGs) and the senescence-associated gene Hexokinase-1, which normally activate during the final stages of leaf senescence [158]. This suppression prevents cellular self-digestion, preserving structural and physiological function.

### 6.2. Water Relations and Structural Integrity

Melatonin maintains water balance and structural rigidity, preventing post-harvest defects such as “neck bending”. MT improves water uptake by regulating aquaporin expression, particularly Plasma Membrane Intrinsic Proteins (PIPs) and Tonoplast Intrinsic Proteins (TIPs) [159]. This increases cell membrane water permeability and hydraulic conductivity, preventing vascular blockage and wilting in cut stems [160]. MT promotes lignin biosynthesis in vascular tissues [161], Lignin is essential for maintaining cell wall rigidity and preventing neck bending, a common defect in large, heavy cut flowers.

### 6.3. Disease Resistance

MT displays antifungal and disease-protective properties. Studies have shown that MT protects Gerbera (*Gerbera jamesonii*) cut flowers against gray mold (*Botrytis cinerea*), a major cause of post-harvest decay [150]. This biotic resistance is further demonstrated in *Lilium* spp., where exogenous MT treatment significantly alleviates leaf blight caused by *Botrytis elliptica*. Transcriptomic profiling coupled with KEGG pathway enrichment analyses indicate that MT orchestrates a coordinated defense response through the activation of genes associated with plant–pathogen interactions, plant hormone signal transduction, and phenylpropanoid biosynthesis. Notably, the pronounced differential expression of genes within the MAPK signaling cascade highlights melatonin’s role as a master regulator of stress-signaling networks. Through the activation of MAPK pathways and phenylalanine metabolism, MT strengthens both physiological and molecular defense mechanisms in *Lilium*, underscoring its potential as a strategic target for molecular breeding programs aimed at achieving durable disease resistance [162,163]. These disease-resistance effects are often mediated through MT’s regulatory influence on other defense hormones, including Salicylic Acid (SA), Jasmonic Acid (JA), and Nitric Oxide (NO). These signaling pathways collectively enhance the plant’s innate immune system [88,163,164].

## 7. Practical Applications and Limitations

While the molecular efficacy of phytomelatonin (MT) is well-established, its translation into commercial floriculture and nursery management relies on optimizing delivery systems and strictly adhering to dose-dependent protocols. The application strategy must be tailored to the specific developmental stage of the plant and the type of stress encountered.

### 7.1. Efficacy of Application Methods

The exogenous application of MT is typically achieved through several primary methods: seed and bulb priming, foliar spraying, and root drenching. Each method triggers distinct physiological cascades with varying durations of efficacy.

The selection of a melatonin application method in horticultural practice is dictated by the specific production objective and the anatomical constraints of the target species. Foliar application is frequently preferred in large-scale greenhouse production due to its ease of integration into existing irrigation/misting systems and its rapid efficacy in reversing sudden environmental stress (e.g., sudden heat waves) [89,98]. Conversely, root drenching and bulb soaking are preferred during the propagation phase; although they require higher volumes of melatonin, they ensure a more stable, systemic distribution that is essential for long-term physiological changes like adventitious rooting or bulb maturation [19,84]. Seed and bulb priming are considered the most cost-effective methods for high-volume nurseries, as they require minimal concentrations to induce ‘stress-memory’ in the early developmental stages [35,121].

Seed Priming (Hormopriming): Seed priming involves soaking seeds in an MT solution prior to germination. This method initiates the “pre-germinative metabolism” without radical protrusion. MT penetrates the seed coat, upregulating the antioxidant systems (SOD, CAT) and DNA repair mechanisms responsible for maintaining genome integrity during imbibition. This is the most effective method for establishing uniform crop stands in bedding plants (e.g., *Petunia*, *Tagetes*). Research indicates that priming provides a “memory effect,” where the seedlings retain enhanced stress tolerance weeks after germination, a phenomenon linked to epigenetic modifications [165]. It is particularly superior for overcoming dormancy in species with low germination vigor [166].

Foliar Spraying: Foliar application relies on the penetration of MT through the cuticle and stomata. Once inside the mesophyll, it is rapidly metabolized or transported via the phloem.

This is the industry standard for immediate stress relief (curative action). It is highly effective for mitigating acute aerial stresses such as heat waves or high light intensity in greenhouse production. In cut flower production (e.g., *Tuberose*), foliar sprays applied pre-harvest significantly improve subsequent vase life by loading the tissues with antioxidants before severing the stem [98]. However, its effects are often transient, requiring repeated applications,

Root Drenching: MT applied to the rhizosphere is absorbed by the roots and translocated systemically to the shoots via xylem transport (acropetal movement). Drenching is superior for combatting soil-borne stresses such as salinity, heavy metal toxicity, or root rot pathogens. In woody ornamentals and propagation cuttings, drenching ensures prolonged exposure of the basal tissues to MT, regulating root architecture more effectively than foliar sprays [69]. However, this method requires larger volumes of solution, increasing input costs.

### 7.2. Dose-Dependency: The Hormetic Effect

A critical limitation in the commercialization of MT is its biphasic dose–response, scientifically known as the Hormetic Effect. MT acts as a biostimulant at low concentrations but can become inhibitory or toxic at supra-optimal levels.

The Optimal Window (Promotive Phase):

Generally, concentrations in the range of 10 to 100 µM are considered optimal for most ornamental species. Within this window, MT functions synergistically with auxins to promote cell expansion, root differentiation, and photosynthetic efficiency. For example, in *Limonium bicolor*, low doses effectively promoted seed germination and seedling growth under salt stress [125].

The observed variability in melatonin’s effectiveness across different ornamental crops is primarily driven by three species-related factors. First, the basal endogenous level of melatonin varies significantly; species with naturally lower concentrations often exhibit a more pronounced response to exogenous treatment as they reach the signaling threshold more quickly [44,45]. Second, anatomical differences, such as cuticle thickness and stomatal density, dictate the efficiency of foliar uptake, requiring adjusted concentrations for waxy-leaved vs. thin-leaved ornamentals to ensure the molecule reaches the mesophyll [89,98]. Finally, the metabolic rate, the speed at which a species converts melatonin into intermediates like 2- or 3-hydroxy-melatonin, determines the duration of the biostimulant’s protective effect [42]. These factors explain why woody ornamentals, such as *Punica granatum* [84] or *Prunus* species [83], typically tolerate higher doses (up to 200 µM) than sensitive herbaceous seedlings.

The Inhibitory Window (Toxic Phase):

Concentrations exceeding 500–1000 µM frequently result in growth inhibition [167]. The inhibition is often attributed to “auxin overdose” symptoms. Since MT and indole-3-acetic acid (IAA) share biosynthetic pathways and structural homology, excessive MT can lead to supra-optimal auxin signaling, inducing ethylene production which inhibits root elongation and causes leaf epinasty [168]. While MT is an antioxidant, extremely high intracellular concentrations can paradoxically disrupt cellular redox homeostasis. By scavenging too many ROS, MT may interfere with essential ROS signaling (which is necessary for cell division), or even induce a pro-oxidant state that damages cell membranes, similar to the action of certain herbicides [23]. Growers must perform small-scale trials, as the threshold for toxicity varies by species. For instance, woody ornamentals may tolerate higher doses (200 µM) compared to sensitive herbaceous seedlings (50 µM).

Despite its benefits, the Hormetic Effect presents significant risks that hinder large-scale adoption. At supra-optimal concentrations, melatonin acts as an inhibitory molecule rather than a biostimulant [167]. This is primarily due to its auxin-mimicking activity; excessive melatonin triggers a ‘hormone overdose’ response, stimulating the production of stress-related ethylene, which leads to leaf epinasty and stunted root development [168]. Furthermore, application at high titers can paradoxically induce reductive stress or act as a pro-oxidant [23]. By scavenging ROS too aggressively, melatonin can deplete the low-level ROS signals necessary for fundamental processes like cell elongation and development [45]. These findings highlight a critical gap: the lack of standardized, long-term field trials that account for environmental degradation and potential soil microbial disruption, which remains a major limitation in current research [169].

## 8. Future Perspectives

The elucidation of phytomelatonin’s role as a master regulator has opened new avenues for innovation in ornamental horticulture. However, transitioning from experimental success to standard industry practice requires addressing critical gaps in genetic engineering and commercial formulation.

### 8.1. Genetic Engineering: The Next Frontier

While exogenous application is effective, the future of stress-resilient ornamentals lies in genetic bio-fortification engineering plants to synthesize high levels of endogenous melatonin constitutively or under stress induction. Current research has successfully generated transgenic lines in model species (like Arabidopsis and rice) by overexpressing key biosynthetic genes, particularly Serotonin N-acetyltransferase (SNAT) and Acetylserotonin O-methyltransferase (ASMT). These transgenic plants exhibit “super-survival” traits, including delayed senescence and extreme drought tolerance [169,170].

Application in Ornamentals: The translation of this technology to high-value ornamentals (e.g., *Petunia*, *Chrysanthemum*, turfgrasses) offers immense economic potential. By engineering turfgrass with elevated endogenous melatonin, breeders could develop “low-maintenance” varieties that require significantly less irrigation and fertilizer, aligning with sustainable urban landscaping goals [171]. Beyond overexpression, genome editing tools like CRISPR/Cas9 could be used to knock out the catabolic enzymes (such as Melatonin 2-hydroxylase, M2H) that degrade melatonin. This would effectively increase cellular melatonin titers without introducing foreign DNA, potentially easing regulatory pathways for commercial release [168].

### 8.2. Commercial Formulations and Scalability

A significant bottleneck in current research is the transition from controlled-environment experiments to large-scale field applications. While melatonin has shown consistent efficacy in lab and greenhouse settings, its effectiveness in open-field horticulture is significantly influenced by environmental variables such as UV-induced photodegradation and microbial breakdown in the soil [172,173]. Currently, the majority of research focuses on high-value ornamental species in restricted environments; therefore, the cost-to-benefit ratio for large-scale landscape or field-grown flower production remains under-investigated. To bridge this gap and facilitate commercial adoption, the industry requires a three-tiered approach.

First, research must shift from general efficacy trials to defining precise, species-specific ‘dose–response’ curves. The effects of exogenously applied melatonin are highly dependent on concentration, crop species, and cultivar, with optimal doses varying significantly between greenhouses and open fields [174]. Establishing these specific rates is essential to maximize benefits while ensuring growers avoid the inhibitory risks of the hormetic zone, where higher doses can negatively impact yield [175,176,177].

Second, future research must prioritize the development of stable, agricultural-grade formulations—specifically nano-carriers—to ensure sustained release under variable field conditions [28,151]. The inherent instability of melatonin limits its field effectiveness [178]; however, encapsulating melatonin in biodegradable polymers or mesoporous silica protects the active ingredient from premature breakdown [169,179]. These advanced formulations are a critical step in translating experimental success into standard industry practice [180].

Finally, practical integration protocols must be established that allow melatonin to be co-applied with standard fertilizers or fungicides via existing irrigation systems. This strategy minimizes labor costs while maximizing long-term plant health [181,182]. While melatonin can act as a ‘safener’ against chemical stress, some studies indicate potential antagonistic effects with certain fertilizers like urea [183,184]. Therefore, defining compatible co-application protocols for foliar sprays or soil drenching is crucial for the adoption of melatonin in sustainable commercial agriculture [182].

## 9. Conclusions

Phytomelatonin represents a paradigm shift in ornamental plant physiology. It is not merely an antioxidant but a pleiotropic orchestrator of development, capable of enhancing root architecture, intensifying floral pigmentation, and extending the critical post-harvest window of cut flowers. By integrating melatonin-based protocols, whether through seed priming, exogenous spraying, or future transgenic breeding, the floriculture industry can achieve a dual goal: maximizing esthetic quality while minimizing resource inputs (water, synthetic pesticides). As climate variability challenges traditional horticulture, melatonin stands out as a sustainable, potent tool for securing the resilience and beauty of the global ornamental market.

## Figures and Tables

**Table 2:** Summary of recent studies on melatonin-mediated abiotic stress tolerance and growth regulation in ornamental species.

Plant Species	Stress/Growth Condition	Concentration Applied	Method of Application	Observed Effects	Author(s)
*Calendula officinalis*	Normal Growth	100–150 µM	Foliar Spray	Increased chlorophyll a/b & carotenoids; improved photosynthetic efficiency.	[89]
*Calendula officinalis*	Salt Stress (42–128 mM)	100 µM (+bacteria)	Combined (Foliar/Soil)	Enhanced NPK uptake; reduced Na^+^/Cl^−^; improved membrane stability.	[90]
*Capsicum annuum*	Cadmium (Cd) Stress	50 µM (±Trehalose)	Foliar Spray	Synergistic reduction in ROS; upregulation of defense genes.	[91]
*Chrysanthemum*	Heat Stress	200 µM	Foliar Spray	Reduced oxidative injury; regulated heat-shock gene networks.	[92]
*Chrysanthemum morifolium*	Drought Stress	100 µM	Foliar Spray	Improved photosynthesis; lower MDA; higher antioxidant enzymes.	[86]
*Clematis* spp.	Waterlogging Stress	50–200 µmol·L^−1^	Root Drench/Spray	Reduced H_2_O_2_; boosted APX, POD, SOD; modulated defense TFs.	[93]
*Cymbidium* spp.	Abiotic Stress	Endogenous Study	N/A (Genetic Analysis)	Identification of SNAT, COMT, TDC genes; upregulation of endogenous MT.	[54]
*Dianthus caryophyllus*	Heat Stress	5–10 mM	In vitro Media	Upregulated HSP genes; increased biomass & chlorophyll.	[94]
(*Gerbera jamosonii* L. cv. *Yunnanhong*)	Salt Stress (150 mM)	0.2 mM	Foliar Spray	Enhanced K^+^ uptake; improved growth & pigments.	[29]
*Gladiolus grandiflores* (sword lily)	Salt Stress (5 dS/m)	0.6 mM	Foliar Spray	Higher antioxidants (3×); reduced MDA & H_2_O_2_.	[95]
*Lonicera japonica*	Salt Stress (150 mM)	60 µM	Root Drench/Spray	Upregulated PAL & CHS; activated transcription factors.	[96]
*Matthiola incana*	Cadmium Stress	100 µM (±H_2_S)	Foliar Spray	Synergistic effect with H_2_S; improved photosynthesis.	[97]
*Polianthes tuberosa* L.	Arsenic Stress (50 μM)	100 µM	Foliar Spray	Improved growth; reduced ROS; higher antioxidants.	[98]
*Prunus avium* × *P. cerasus*	Propagation (Rooting)	0.05–10 µM	In vitro Media	0.5–1 µM promoted rooting; high doses (5–10 µM) inhibited it.	[83]
*Ranunculus asiaticus*	Salinity stress (4.5–5.5 dS·m^−1^)	200 µM	Foliar spray	↑ Chlorophyll, carotenoids, RWC, proline, and POD; ↓ Na^+^ content and electrolyte leakage; delayed flowering.	[99]
*Ranunculus asiaticus*	Drought Stress & Normal	200 µM	Foliar spray	Enhanced growth (biomass/leaf area); 21-day earlier flowering; ↑ proline and POD; ↓ electrolyte leakage.	[100]
*Rhododendron maculiferum*	Flowering Regulation	300 µmol·L^−1^	Foliar Spray	Accelerated flowering (32 days earlier); increased GA_3_/IAA; lower ABA.	[101]
*Rhododendron*	Heat stress (35–40 °C)	200 µM	Foliar Spray	↑ Electron transport rate; ↑ Rubisco activity and ATP content; regulated expression of RhRbsA.	[102]
*Rhododendron simsii*	Cadmium (Cd) Stress	200 µM	Foliar Spray	↑ Net photosynthetic rate (P_n_) and F_v_/F_m_; protection of PSI and PSII (donor and receptor sides); ↑ SOD and POD; ↓ MDA.	[103]
*Zinnia elegans*	Drought (20% FC)	1.0 mM	Foliar Spray	Increased CO_2_ assimilation & stomatal conductance; improved RWC.	[104]

**Table 3:** Post-harvest quality and vase life.

Plant Species	Condition/Pathogen	Concentration Applied	Method of Application	Observed Effects	Author(s)
*Alstroemeria* ‘*Amatista*’	Vase Life/Senescence	100–200 µM	Vase Solution	Extended vase life (13→21 days); higher anthocyanin, PAL, PPO.	[145]
*Alstroemeria* ‘*Amatista*’	Vase Life/Senescence	50 µM (+Putrescine)	Pre-harvest Spray	Extended vase life (16→23 days); downregulated CHL & ACO genes.	[146]
*Snapdragon* (*Antirrhinum majus* L.)	Postharvest Quality	200 µmol·L^−1^	Vase Solution	Increased stem/floret size; improved flowering under WRB light.	[147]
*Chrysanthemum*	Postharvest Senescence	5 µM	Vase Solution	Improved water balance; reduced LOX activity; longer vase life.	[148]
*Dianthus caryophyllus*	Postharvest Senescence	0.1 mM	Vase Solution	Extended vase life by 10 days; better membrane stability.	[74]
*Gardenia jasminoides*	Dark-induced Senescence	1.0 mM	Post-harvest Dip	Delayed yellowing; regulated hormone metabolism.	[149]
*Gerbera jamesonii*	Gray Mold (*Botrytis*)	200 µM	Foliar Spray (Pre)	Reduced disease severity; increased lignin & PAL.	[150]
*Gerbera jamesonii*	Postharvest Senescence	0.1–0.5 mM	Nano-formulation (Dip)	Extended vase life; higher CAT activity; reduced oxidative stress.	[28]
*Gerbera jamesonii*	Postharvest Senescence	20 mM (MT-CuNPs)	Foliar app.	Extended vase life (6 days); increased xylem thickness.	[151]
*Hemerocallis fulva*	Postharvest Senescence	120 µM	Vase Solution	Lower bacterial growth; vase life 7→12 days.	[152]
*Paeonia lactiflora*	Postharvest Senescence	50 µmol·L^−1^	Vase Solution	Larger flowers; lower MDA; higher SOD & CAT.	[153]
*Paeonia suffruticosa*	Postharvest Senescence	0.4 mg·L^−1^	Vase Solution	Prolonged vase life; better water balance & protein content.	[154]
*Peony ‘Bartzella’*	Flower Opening	100 µM	Vase Solution	Delayed early opening; modulated ethylene-dependent senescence.	[155]
*Pilea* spp. *P. cadierei*, *P. involucrata*, and *P. mollis*	Dark-induced Senescence	50–150 µmol·L^−1^	Foliar Spray (Pre-storage)	Better storage quality; increased pigments & leaf area.	[156]

## Data Availability

No new data were created in this study.
